# Periscope Proteins are variable-length regulators of bacterial cell surface interactions

**DOI:** 10.1073/pnas.2101349118

**Published:** 2021-05-31

**Authors:** Fiona Whelan, Aleix Lafita, James Gilburt, Clément Dégut, Samuel C. Griffiths, Huw T. Jenkins, Alexander N. St John, Emanuele Paci, James W. B. Moir, Michael J. Plevin, Christoph G. Baumann, Alex Bateman, Jennifer R. Potts

**Affiliations:** ^a^Department of Biology, The University of York, YO10 5DD York, United Kingdom;; ^b^European Molecular Biology Laboratory, European Bioinformatics Institute, Wellcome Genome Campus, CB10 1SD, United Kingdom;; ^c^Department of Chemistry, The University of York, YO10 5DD York, United Kingdom;; ^d^Astbury Centre for Structural Molecular Biology, The University of Leeds, LS2 9JT Leeds, United Kingdom

**Keywords:** protein structure, bacterial surface proteins, multidomain proteins

## Abstract

Changes at the cell surface enable bacteria to survive in dynamic environments, such as diverse niches of the human host. Here, we reveal “Periscope Proteins” as a widespread mechanism of bacterial surface alteration mediated through protein length variation. Tandem arrays of highly similar folded domains can form an elongated rod-like structure; thus, variation in the number of domains determines how far an N-terminal host ligand binding domain projects from the cell surface. Supported by newly available long-read genome sequencing data, we propose that this class could contain over 50 distinct proteins, including those implicated in host colonization and biofilm formation by human pathogens. In large multidomain proteins, sequence divergence between adjacent domains appears to reduce interdomain misfolding. Periscope Proteins break this “rule,” suggesting that their length variability plays an important role in regulating bacterial interactions with host surfaces, other bacteria, and the immune system.

## Significance

The structure of single and tandem SHIRT domains from the streptococcal surface protein Sgo_0707 were determined. In conjunction with biophysics and molecular dynamics simulations, the results show that the observed gene length variation would result in differential projection of the host ligand binding domain on the bacterial cell surface. An analysis of long-read DNA sequence data reveals many other repetitive bacterial surface proteins that appear to undergo gene length variation. We propose that these variable-length “Periscope Proteins” represent an important mechanism of bacterial cell surface modification with potential roles in infection and immune evasion.

Bacteria encounter complex and dynamic environments, including within human hosts, and have thus evolved various mechanisms that enable a rapid response for survival within, and exploitation of, new conditions. In addition to classical control by regulation of gene expression, bacteria exploit mechanisms that give rise to random variation to facilitate adaptation [e.g., phase and antigenic variation (1)]. In Gram-positive and Gram-negative human pathogens, DNA inversions (2, 3), homologous recombination (4), DNA methylation (1), and promoter sequence polymorphisms (5) govern changes in bacterial surface components, including capsular polysaccharide and protein adhesins, which can impact bacterial survival and virulence in the host (1, 6). Many of these mechanisms are very well studied and widespread across bacteria.

A less well-studied mechanism is length variation in bacterial surface proteins. Variability in the number of sequence repeats in the Rib domain (7)–containing proteins on the surface of Group B streptococci has been linked to pathogenicity and immune evasion (8). The repetitive regions of the *Staphylococcus aureus* surface protein G (SasG) (9) and *Staphylococcus epidermidis* SasG homolog, Aap (10), also demonstrate sequence repeat number variability. In SasG, this variability regulates ligand binding by other bacterial proteins in vitro (11) in a process that has been proposed to enable bacterial dissemination in the host. Variations in repeat number have also been noted in the biofilm forming proteins Esp from *Enterococcus faecalis* (12) and, more recently, CdrA from *Pseudomonas aeruiginosa* (13). High DNA sequence identity in the genes that encode these proteins is likely to facilitate intragenic recombination events that would lead to repeat number variation (14) and, in turn, to protein sequence repetition. However, such sequence repetition is usually highly disfavored in large multidomain proteins (15), so its existence in these bacterial surface proteins suggests that protein length variation provides an evolutionary benefit. SasG, Aap, and Rib contain N-terminal host ligand binding domains and C-terminal wall attachment motifs; thus our recent demonstration that the repetitive regions of both SasG (16) and Rib (17) form unusual highly elongated rods suggests that host-colonization domains will be projected differing distances from the bacterial surface.

Here, we show that repeat number variation in predicted bacterial surface proteins is more widespread and we characterize a third rod-like repetitive region in the *Streptococcus gordonii* protein (Sgo_0707) formed by tandem array of Streptococcal High Identity Repeats in Tandem (SHIRT) domains. Thus, we propose a growing class of “Periscope Proteins,” in which long, highly similar DNA repeats facilitate expression of surface protein stalks of variable length. This mechanism could enable changes in response to selection pressures and confer key advantages to the organism that include evasion of the host immune system (8) and regulation of surface interactions (11) involved in biofilm formation and host colonization.

## Results

### Defining the Structural Repeats of Sgo_0707 from *S. gordonii*.

**Fig. 1.**
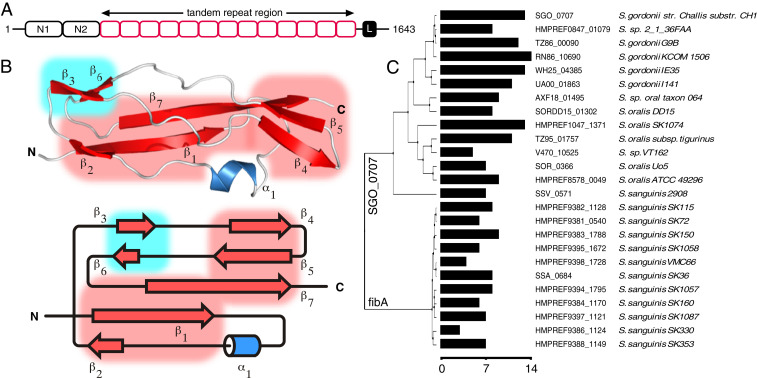
Close homologs of A8AW49 contain variable numbers of repeats that each form the “SHIRT” fold. (*A*) Schematic of Sgo_0707 showing the N-terminal adhesin domains N1 and N2, tandem repeat (SHIRT) domains (red) and C-terminal LPXTG cell-wall crosslinking motif (black box “L”). (*B*) Structure of Sgo_R10 and topology; sheets S1 (red) and S2 (blue) are highlighted in boxes. (*C*) A phylogenetic tree of Sgo_0707 homologs (>70% identity) identified using PHMMER (61) from ENSEMBL bacterial genomes using the N-terminal domain sequence (residue range 1 through 450 for Sgo_0707) and containing the LPXTG cell wall anchor motif at their C termini. The number of SHIRT domain repeats in the stalk of each protein is shown as a bar plot with scale bar below.

Having revealed the unusual repetitive, rod-like characteristics of both SasG (16) and Rib (17) in our previous studies, we used bioinformatic approaches to search for other cell-wall attached bacterial proteins with similar domain architectures. A8AW49 (herein Sgo_0707) encoded by the gene *Sgo_0707* from *S. gordonii* (Fig. 1*A*) has a C-terminal wall attachment motif, homologs with repeat number variation, and a structurally defined two-domain N terminus (N1-N2; residues 36 through 458, Protein Data Bank [PDB]: 4igb) that is proposed to be involved in collagen binding (18). As the repeats had no Pfam definition, we called the putative domain “SHIRT” (Fig. 1*B*). *S. gordonii* is a member of the *S. sanguinis* group of viridans streptococci (19) and is a common colonizer of the oral cavity. It is a pioneer organism in the establishment of dental plaque (20) and also implicated in infective endocarditis (21).

Defining the structural, rather than sequence, repeat boundaries in repetitive bacterial proteins is challenging. For Sgo_0707 the T-REKS server (22) predicts a repeat frame of 460 through 543 and 13 repeats of 84 through 90 residues. A construct based on the second repeat (residues 544 through 627; Δ*N*-Sgo_R2) was folded (*SI Appendix*, Fig. S1*A*) and solved to 0.95 Å resolution using X-ray crystallography (*SI Appendix*, Fig. S1*A*, Inset and Table 1) utilizing ab initio molecular replacement (MR) with ideal fragments (23). Based on the significant truncation of the N-terminal β-strand (*SI Appendix*, Fig. S1*A*, Inset), we hypothesized that shifting the frame of the repeat by seven residues toward the N terminus of Sgo_0707 would complete the fold. Sgo_R3 (residues 621 through 705) and Sgo_R10 (residues 1211 through 1299) based on this new definition were thus expressed and purified. They were found to have significantly higher melting temperatures (*T*_m_) than the N-terminally truncated Sgo_R2 (Δ*N*-Sgo_R2, 55.9 °C; Sgo_R3, 75.7 °C; Sgo_R10, 75.9 °C; *SI Appendix*, Fig. S1*B*).

**Table 1.** Crystallographic data collection and refinement statistics

	Δ*N*-Sgo_R2	Sgo_R10	Sgo_R3-4
Data collection statistics			
Wavelength (Å)	0.85	0.78	0.976
Space group	*P*2_1_2_1_2_1_	*P*2_1_2_1_2	*P*2_1_
Cell dimensions			
a, b, c (Å)	21.4, 40.8, 82.5	65.4, 48.0, 48.4	24.1, 37.2, 101.1
β (°)	90.0	90.0	95.8
Resolution limits (Å)*	82.5–0.95 (0.96–0.95)	38.9–0.82 (0.85–0.82)	37.2–1.35 (1.37–1.35)
No. reflections			
Total*	281,952 (12,914)	292,386 (24,481)	157,956 (7,439)
Unique*	44,546 (2,604)	147,184 (11,281)	39,416 (1,911)
*R*_merge_*^,^ ^†^	0.034 (0.681)	0.045 (2.057)	0.066 (0.960)
Mean *I*/σI*	24.8 (2.3)	28.3 (0.3)	8.9 (1.4)
Half-set correlation CC (1/2)*	0.999 (0.839)	1.000 (0.239)	0.998 (0.562)
Wilson B-factor (Å^2^)	8.9	10.8	10.8
Completeness (%)*	95.2 (75.8)	96.8(76.5)	99.9 (99.6)
Redundancy*	6.3 (5.0)	2.0(1.9)	4.0 (3.9)
Refinement statistics			
Resolution (Å)*	43.2 (0.95)	38.9 (0.82)	34.9 (1.35)
No. of reflections			
Working	42,240	144,297	37,511
Free	2,193	1,454	1,892
*R*_work_/*R*_free_ (%)	12.5/13.3	14.7/16.2	13.4/17.0
Rms from ideality			
Bond length (Å)	0.017	0.017	0.023
Angles (°)	1.96	1.48	2.15
No. of atoms			
Protein	661	1,442	1,404
Ligand/ion	—	15	33
Water	104	247	200
Average B (Å^2^)			
Protein	14.9	18.1	18
Ligand/ion	—	19.7	22.4
Water	28.3	27.8	34.0
Ramachandran angles (%)			
Favored	98.0	98.15	97.0
Allowed	2.0	1.85	3.0
Outliers	0.0	0.0	0.0

* Values in parentheses are for the highest resolution shell.

^†^ *R*_merge_ = Σ|I - <I>|/ΣI.

The structure of Sgo_R10 (Fig. 1*B*) was solved at 0.82 Å resolution using MR with the structure of Δ*N*-Sgo_R2 for phasing; the data collection and refinement statistics are summarized in Table 1. The model confirms that SHIRT has an α/β fold organized around a single α-helix and two distinct β-sheets (Fig. 1*B*). Fig. 1*A* shows a schematic of Sgo_0707 based on the structural boundaries of the repeats; *SI Appendix*, Fig. S1*C* shows the high level of protein sequence identity (82 to 100%) between adjacent SHIRT domains. Comparison of *Sgo_0707* genes from different bacterial strains, including the homologous protein fibA from *S. sanguinis*, shows a high variability in the number of SHIRT domain repeats forming the stalk, ranging from 3 to 14 copies (Fig. 1*C*). SHIRT domains are found in many other proteins, often in tandem array (*SI Appendix*, Fig. S2).

### Tandemly Arrayed Sgo_0707 SHIRT Domains Form an Extended Rod-like Structure.

**Fig. 2.**
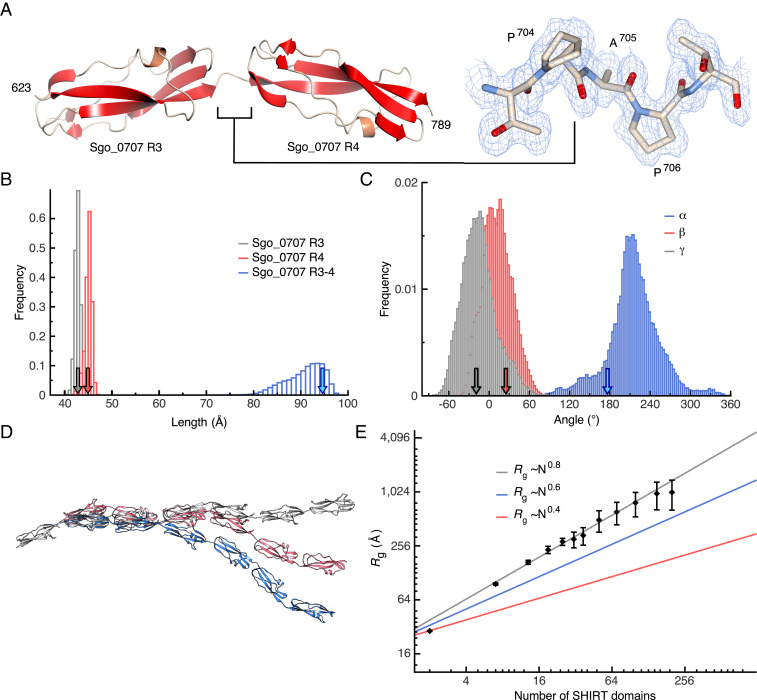
The tandem SHIRT repeat Sgo_R3-4 adopts an anisotropic (head-to-tail) structure with limited interdomain “bend” but significant “twist.” (*A*) The structure of Sgo_R3-4 (*Left*) and connecting short, well-ordered Pro-Ala-Pro interdomain linker; alternate conformers of P704 are included (electron density 2mF_o_-DF_c_ map contoured at 0.1182 electrons/Å^3^ blue chickenwire, *Right*). (*B*) Frequency of domain lengths and (*C*) interdomain angles (*SI Appendix*, Fig. S5*A*) over a 0.8 μs all-atom, fully solvated molecular dynamics simulation of Sgo_R3-4 at 303 K. The ends of the domains Sgo_R3, Sgo_R4, and Sgo_R3-4 and linker are identified by the Cα atoms of residues T623–P704, T707–A789, and T627–A789, respectively; arrows show the value of the distances in the crystal structure. (*D*) Models of seven tandemly arrayed domains based on the α, β, γ angles in *C*. (*E*) Scaling of *R*_g_ of simulated model SHIRT constructs with increasing number of domains (or, equivalently, amino acids) compared with denatured and native proteins (approximated by blue and red lines, respectively). Error bars are SDs over 100 models generated.

A tandem domain construct (Sgo_R3-4; residues 621 through 789) was crystallized, and the structure was solved via MR using the Δ*N*-Sgo_R2 model; data collection and refinement statistics are summarized in Table 1. The structure (Fig. 2*A*) reveals two complete domains with a very short (Pro-Ala-Pro) linker (*SI Appendix*, Fig. S1 *C* and *D*); the structure of Sgo_R3-4 is ordered throughout (residues 623 through 789). Each domain adopts the SHIRT fold, and the interdomain interface is limited; this was confirmed by comparing the *T*_m_ of Sgo_R3 (75.7 °C) and Sgo_R3-4 (76.6 °C; *SI Appendix*, Fig. S3). The similarity of unfolding curves for single and double SHIRT constructs suggests that the two domains in the tandem construct unfold independently. Small angle X-ray scattering (SAXS) analysis substantiates the anisotropic head-to-tail domain arrangement in solution (*SI Appendix*, Fig. S4*A*). Notably, there is a significant twist between domains when viewed along the long axis of the molecule.

Molecular dynamics (MD) simulations of the Sgo_R3-4 construct show that individual domains are particularly stable (rmsd <1.5 Å for Cα atoms) over the length of the trajectory (0.8 μs); their individual length is conserved during the simulation and the length of Sgo_R3-4 fluctuates only moderately around 97 Å (Fig. 2*B*). The distributions of α, β, γ interdomain (17) angles (*SI Appendix*, Fig. S5*A*) observed in the simulations of the Sgo_R3-4 construct (Fig. 2*C*) were used to generate models of longer constructs (Fig. 2*D*). The radius of gyration (*R*_g_) of the simulated constructs increases following the relation $R_{g}\propto N^{\nu }$, where ν is the Flory exponent and describes the increase in size of a polymer (protein) made of N monomers (amino acids). Such an exponent is ∼0.6 for denatured proteins and ∼0.4 for folded ones (24). Polymers formed of sequential SHIRT domains are highly extended; *R*_g_ scales with the number of domains (or equivalently, amino acids) with an exponent ν∼0.8 (Fig. 2*E*), which is remarkable given the width of the distribution of the angles describing the mutual orientation of adjacent domains (Fig. 2*C*).

**Fig. 3.**
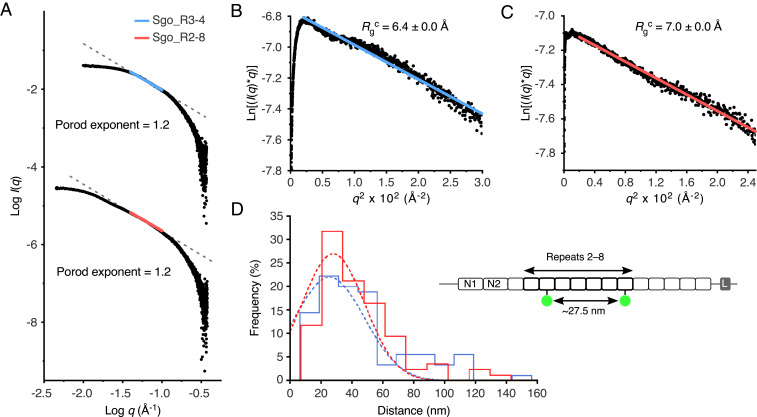
Tandemly arrayed SHIRT domains form a highly elongated rod. (*A*) Porod plots from SAXS analysis of Sgo_R3-4 (*Top*) and Sgo_R2-8 (*Bottom*). Porod exponents were calculated from the negative slope of the linear region of the data (R3-4, blue, *R*^2^ = 1.0; R2-8, red, *R*^2^ = 1.0). (*B*) Modified Guinier region for Sgo_R3-4 with cross-sectional radius of gyration (*R*_g_^c^, annotated) calculated from fitted region (blue line, *R*^2^ = 0.98, q*R_g_ limits = 0.3 to 1.1). (*C*) Modified Guinier region for Sgo_R2-8 with *R*_g_^c^ calculated from fitted region (red line, *R*^2^ = 0.99, q*R_g_ limits = 0.3 to 1.0. (*D*) SHRImP-TIRFM determination of the interdye distances for AF488-labeled Sgo_R2-8^S666C/S1086C^ (*Schematic Inset*) immobilized on 2 µg/mL (blue) or 20 µg/mL (red) poly-D-lysine-treated quartz surfaces. Color-coded, dashed lines indicate Gaussian fits to the histograms for the 2 µg/mL (*n* = 90, *R*^2^ = 0.86) and 20 µg/mL (*n* = 85, *R*^2^ = 0.90) poly-D-lysine-treated quartz surfaces (mean ± SE = 25.5 ± 2.39 nm and 27.8 ± 2.21 nm, respectively).

To assess the elongation of Sgo_0707 in solution, we collected SAXS data for constructs comprising two (Sgo_R3-4) and seven (Sgo_R2-8) tandemly arrayed SHIRT domains (Fig. 3 and *SI Appendix*, Fig. S4). Both Sgo_R3-4 and Sgo_R2-8 are monomeric in solution, eluting as monodisperse peaks from size exclusion chromatography (SEC) columns (*SI Appendix*, Fig. S4 *A* and *B*, Inset). Both the crystal structure of Sgo_R3-4 (Fig. 2*A*) and an elongated model for Sgo_R2-8 (*SI Appendix*, Fig. S4*B*) are consistent with the SAXS data measured in solution (model:data fits of χ^2^ = 1.1 and χ^2^ = 1.2, respectively; *SI Appendix*, Fig. S4 *A* and *B* and *Materials and Methods*). Analysis of the data for Sgo_R3-4 and Sgo_R2-8 results in equal Porod exponents (1.2, Fig. 3*A*), as well as similar radii of gyration of a cross-section (*R*_g_^c^) values (R3 through 4 = 6.4 ± 0.0 Å; R2 through 8 = 7.0 ± 0.0 Å; Fig. 3 *B* and *C*). Consistent with the models (Fig. 2*D*), the *D*_max_ that was determined using SAXS scales with the number of domains; Sgo_R3-4 exhibits a *D*_max_ of 107 Å and Sgo_R2-8 has a *D*_max_ of 371 Å (*SI Appendix*, Fig. S4 *A* and *B*). Therefore, while displaying a much larger intraparticle maximum dimension, the Sgo_R2-8 construct has a comparable shape and *R*_g_^c^ to Sgo_R3-4.

In our previous study (17), we observed elongation in two-domain Rib constructs with rotation of angle α, while angles β and γ were smaller. We conducted the same analysis for Sgo_0707 constructs, based on fitting to our experimental SAXS data (*SI Appendix*, Fig. S5). As with Rib, MD simulations of the two-domain Sgo_R3-4 construct show that a range of α angles give good fits to the observed SAXS data, while β and γ are restricted to a narrow range, consistent with an elongated conformation (*SI Appendix*, Fig. S5*B*). For fitting of MD simulations of the 7-domain Sgo_R2-8 construct to SAXS data, we observed that longer end-to-end distances improved the quality of the fit (*SI Appendix*, Fig. S5*C*). Taken together, these data are consistent with multiple tandemly arrayed SHIRT domains from Sgo_0707 behaving as an elongated, rod-like particle.

To further assess the elongation of multidomain SHIRT constructs, we used a high-resolution single-molecule technique [SHRImP (16)] to measure the intramolecular distance between two Alexa Fluor 488 (AF488) dyes covalently attached to cysteine residues engineered at specific sites in Sgo_R2-8 (S666C and S1086C). If the extended interdomain topology observed in the two-domain construct is maintained, a distance of 24.1 nm between the mean dye positions (*SI Appendix*, Fig. S6*A*) is predicted by using the distance between the two cysteine residues (Fig. 3 *D*, Inset) and simulating the increased volume accessible to each dye due to the chemical linker (25). AF488-labeled Sgo_R2-8^S666C/S1086C^ was imaged using total internal reflection fluorescence microscopy (TIRFM) using two different poly-D-lysine concentrations to coat the slides. Both surface treatments effectively immobilized AF488-labeled Sgo_R2-8^S666C/S1086C^, and each SHRImP-TIRFM histogram had a single peak consistent with monodispersity. The measured interdye distances were 25.5 nm and 27.8 nm (Fig. 3*D*) with 2 μg/mL and 20 μg/mL poly-D-lysine concentration, respectively, and increased to 29.8 nm and 33.0 nm, (*SI Appendix*, Fig. S6*B*), respectively, when measurements were performed in the presence of 100-fold molar excess of unlabeled Sgo_R2-8 (termed “blocking” protein). This implies the rod-like conformation of Sgo_R2-8 protein is malleable, adopting, compared with solution, a slightly more elongated state when on a surface which is accentuated (∆x = 4 to 5 nm) at high-protein concentrations that presumably favor lateral protein–protein interactions.

### Large-Scale Identification of Potential Periscope Proteins in Bacterial Genomes.

**Fig. 4.**
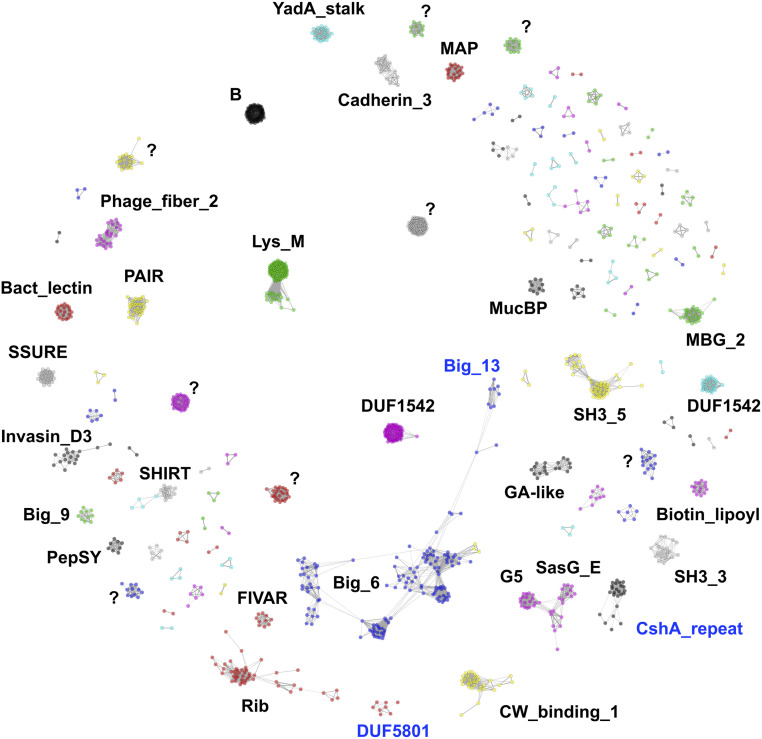
Sequence clustering of long tandem highly identical repeats identified across proteins of the NCTC3000 genomes. The largest clusters are annotated using Pfam (Pfam IDs are found in Dataset S1). Clusters with unknown Pfam classification are marked with “?”. New protein domain families built from sequence clusters into Pfam are highlighted in blue.

We call SasG, Rib, and Sgo_0707 “Periscope Proteins” due to their having variable-length stalk-like regions that define the distance that the N-terminal functional domain projects from the bacterial surface. We conducted an unbiased identification of other potential Periscope Proteins from the NCTC3000 bacterial genomes: an ongoing project lead by the Wellcome Sanger Institute (UK) that provides high quality annotated genome assemblies for 3,000 bacterial strains from Public Health England's National Collection of Type Cultures (NCTC) using the long-read PacBio technology (https://www.sanger.ac.uk/resources/downloads/bacteria/nctc/). The use of long-reads spanning whole genes overcomes the challenges in the assembly of repetitive genes in bacterial genomes (26), providing reliable numbers of repeats in the assembled genes and associated proteins and allowing the identification of length variability in Periscope Proteins. We identified 1,576 proteins containing long and highly identical repeats (i.e., similar to those forming stalks in Periscope Proteins), out of a total of 2.5 million proteins in the NCTC3000 data set, from different species including both Gram-positive and Gram-negative bacteria. Clustering of the repeating sequences reveals most of them are classified into existing Pfam families (Fig. 4 and Dataset S1) that correspond to globular domains, as in known Periscope Proteins, and from a wide range of secondary structure and fold types.

Most of the domain families we find correspond to Immunoglobulin-like beta sandwich folds in the E-set clan (Pfam: CL0159), but we also find domain families with beta grasp folds in the Ubiquitin clan (Pfam: CL0072) and three-helix bundles in the bacterial immunoglobulin/albumin-binding clan (Pfam:CL0598). As expected, the distance between the N and C termini of domain repeats is large, and their termini are oriented close to 180° (3.14 radians; *SI Appendix*, Table S3), enabling linear arrangements of tandem domain repeats. In addition, most interdomain linkers are short (smaller than 5 residues), and some of them are rich in prolines, conferring additional rigidity to the linear domain arrangements. Some exceptions with short intertermini distance and small angles, such as LysM and SH3_3 domains, contain longer and more flexible linkers.

**Fig. 5.**
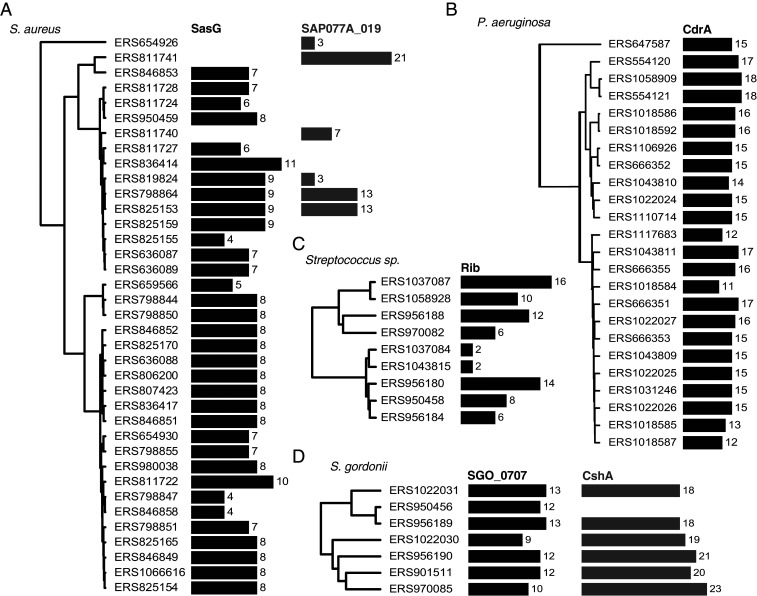
Variation of stalk region repeat numbers in Periscope Proteins. Phylogenetic trees of (*A*) *Staphylococcus aureus*, (*B*) *Pseudomonas aeruginosa*, (*C*) various streptococcal species including *S. agalactiae* and *S. pyogenes*, and (*D*) *S. gordonii* genomes in the NCTC3000 collection, mapped to the number of repeats in stalk regions in Periscope genes; respectively, SasG with G5/E repeats, SAP077A_019 with Big_6 domain repeats, CdrA with MBG_2 domain repeats, surface protein Rib with Rib domain repeats, Sgo_0707 homolog containing SHIRT domain repeats, and surface adhesin CshA with ∼100 residue globular domain repeats of a new Pfam family (CshA_repeat).

We further clustered full-length repetitive proteins and identified a total of 180 unique groups, 84 of which exhibit repeat number variation (Dataset S1). Manual inspection confirmed that 56 of these groups are examples of Periscope Proteins, 30 of which could be assigned to recognizable gene names. We retrieved known Periscope Proteins, such as SasG, Rib, and Sgo_0707 (clusters 4, 25, and 26, respectively), as well as novel ones, for example, CdrA from *Pseudomonas aeruginosa* (27), which has been reported recently to exhibit variability in the size of the repeat region (13), CshA from *S. gordonii* (28), and SAP077A_019 (UniProt D2JAN8) from *S. aureus* (Fig. 5). The majority of confirmed Periscope Proteins (40/56) have at least one Gene Ontology (GO) location term assigned, all of them associating to the cell-wall or bacterial membrane.

**Fig. 6.**
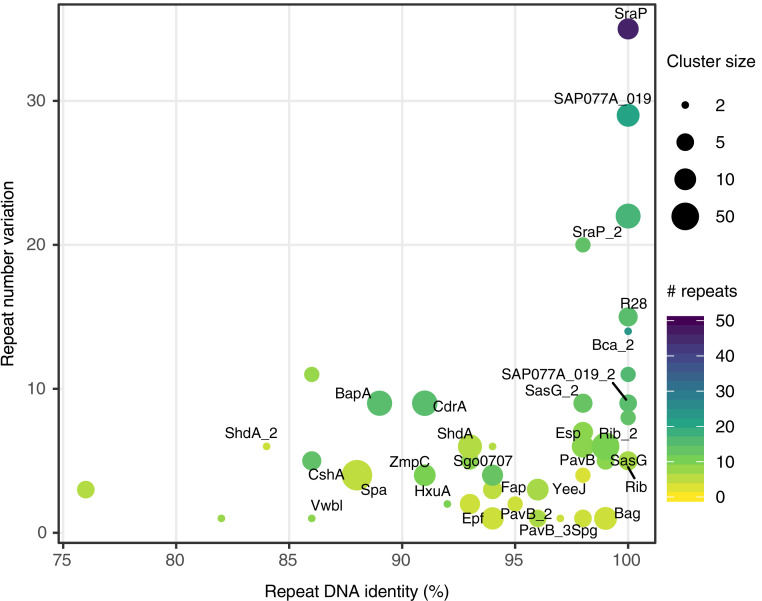
Repeat number variation in Periscope Proteins as a function of repeat sequence identity. The sequence identity of tandem repeats in the genome plotted against the variation in repeat number observed for each Periscope Protein cluster. The repeat number variation is calculated as the difference between the maximum and minimum observed repeat numbers in proteins within each cluster. The maximum number of repeats is shown as a viridis color scale. The repeat DNA identity is calculated as the maximum repeat sequence identity across proteins in each cluster. The number of proteins in each cluster is shown as point size. Names for the most relevant Periscope Protein clusters are shown as labels.

We further find that the number of repeats per gene can be large [>40 in one case (29); Fig. 6], spanning thousands of amino acids, and that variation in the number of stalk repeats can be extreme, increasing the length of the protein by an order of magnitude in some cases. Importantly, we found the most extreme repeat numbers and length variation in proteins with the highest repeat identity (Fig. 6). Furthermore, as shown in Fig. 5, the number of repeats changes drastically even between similar bacterial strains, suggesting a rapid evolutionary rate of repeat number change in Periscope Proteins.

## Discussion

The work presented in this study identifies a class of bacterial surface proteins; through length variation, these proteins are likely to modulate surface interactions. Sgo0707 is the third example of this class for which we have characterized both isolated domains and tandem arrays. SasG, the first example (16), is composed of arrays of E and G5 domains. E domains are unstable in the absence of G5 domains. The interdomain interfaces (E-G5 and G5-E) make very significant contributions to the overall stability of the tandem array of E-G5 domains. G5 domains also have an unusual flat structure rather than a “typical” hydrophobic core. The second example, Rib, is different. We reported the 3D structure of a Rib domain (17), which appears to be an interesting example of domain atrophy from the common Ig-fold. In Rib, there is no evidence for a significant interdomain interface. SHIRT (presented here) is different again. We report the 3D structure of a SHIRT domain which, while also containing β-sheets, has a topology with no obvious relationship to the Ig fold. Like Rib, there is no evidence for significant interdomain interfaces in tandem arrays of SHIRT domains. Rib and Sgo_0707 have relatively short interdomain linkers between Rib and SHIRT domains, respectively, and/or linkers that contain proline residues; both features are likely to contribute to the observed elongation of the tandemly arrayed domains. In addition, due to requirements of forming an elongated array, we predict that, as observed for SasG [PDB: 3TIP; (30)], Rib [PDB: 6S5X; (17)], and Sgo_0707 (Fig. 1), Periscope Proteins will often contain domain folds that locate the N and C termini of the protein sequence at either end of the fold (*SI Appendix*, Table S3). Thus, in Periscope Proteins, we find that different types of domains, when arrayed in tandem, can form an elongated rod that typically serves to project a functional domain distal to the cell surface.

To classify this diverse family of proteins with a memorable analogy, we call them “Periscope Proteins.” We propose that high-sequence similarity at the DNA level enables intragenic recombination events (14) (and thus loss or gain of repeats) with selection pressures resulting in enrichment of bacteria with shorter or longer proteins. Periscope Proteins include proteins implicated in biofilm formation in both Gram-positive and Gram-negative bacteria, for example, SAP077A_019 from *S. aureus* and related proteins in *S. epidermidis*, *Staphylococcus xylosus*, *Staphylococcus capitis*, *Staphylococcus simiae*, and *Staphylococcus warneri*, the *Enterococcus faecalis* protein Esp (12) and related proteins, and CdrA from *Pseudomonas aeruginosa* (13). We expect the total number of Periscope Proteins across bacterial species to be much larger and more widespread than reported in this study. Our knowledge of Periscope Proteins will increase as more long-read sequence data for a diverse set of strains not included in the NCTC3000 collection become available. We suggest that length variation is the main function of the tandemly arrayed regions of Periscope Proteins; however, as some of the domains found in such arrays (such as LysM domains) have had a ligand function assigned, they could play an additional functional role in some contexts.

Large multidomain proteins usually have <50% sequence identity between adjacent domains (15), which has been proposed as an evolutionary strategy to minimize interdomain misfolding (31–33). The existence of Periscope Proteins, which contain tandemly arrayed structured domains with high sequence similarity, confounds this observation. Considering this potential “misfolding problem,” the existence of Periscope Proteins suggests that length variation (driven by the requirement for high identity at the DNA level and thus high protein similarity) is functionally important and confers a significant advantage to the organism. Simply expressing a Periscope Protein (an extreme form of length variation) results in changes in ligand binding by other bacterial surface proteins through spatial competition. For example, expression of Pls (34) and SasG significantly inhibits binding of *S. aureus* to the human plasma proteins fibronectin and fibrinogen, respectively; the latter resulting in altered bacterial colony morphology (35). In both cases, it is suggested that the elongated Periscope Protein is blocking interactions of the host protein with bacterial proteins closer to the cell surface. There is also more direct evidence for the role of length variation in Periscope Proteins under selection pressure. For example, in mice immunized with anti-serum raised against the nine-repeat alpha C, GBS expressing alpha C with one Rib repeat were 100-fold more pathogenic than GBS expressing alpha C with nine Rib repeats (36). It was proposed that the shorter protein is not recognized because it is less exposed. When SasG was expressed on *S. aureus* with differing numbers of repeats, only the longer variants blocked binding of other bacterial surface proteins to their target (11). These authors noted that the ability of a bacterium to detach from a ligand could be important for dissemination in the host. Finally, deletion of CdrA had the most deleterious effect on biofilm formation for *P. aeruginosa* strains expressing the longest CdrA variants (13). Rib repeat number variation within a single strain can be observed within 24 h following inoculation of a mouse with group B streptococci (37), suggesting the potential for dynamic regulation by Periscope Proteins on a physiologically relevant timescale. In summary, surface variation through the Periscope Protein class appears to be a highly distinctive way to alter a variety of interactions linked to colonization and infection.

## Materials and Methods

### Cloning.

The *Escherichia coli* codon-optimized coding sequence for *S. gordonii* strain Challis (substrain DL1) Sgo_0707 residues 544 through 795 (UniProt: A8AW49) was synthesized (Genewiz; *SI Appendix*, Table S2) and the truncated single repeat Δ*N*-Sgo_R2 (amino acids 544 through 627), single repeats Sgo_R3 (aa 621 through 705) and Sgo_R10 (aa 1211 through 1299) (synthesized by Eurofins Genomics), and tandem repeat Sgo_R3-4 (aa 621 through 789) were cloned downstream of a hexahistidine tag and 3C protease specific linker by the In-fusion method (Clontech; primer sequences listed in *SI Appendix*, Table S1). DNA coding for the 7-repeat construct (Sgo_R2-8) comprising the second to eighth SHIRT repeats (aa 537 through 1125) was synthesized (Eurofins Genomics) and inserted by homologous recombination into a modified pBAD vector (pBADcLIC2005), generating a coding sequence comprising GGGFA-Sgo_R2-8-His_10_. Site-directed mutagenesis was used to introduce cysteine mutations in the Sgo_R2-8 construct for fluorescent dye modification at positions S666 and S1086 (Sgo_R2-8^S666C/S1086C^; mutagenesis primer sequences listed in *SI Appendix*, Table S1).

### Protein Expression and Purification.

For Δ*N*-Sgo_R2, Sgo_R3, and Sgo_R3-4, expression was induced in *E. coli* BL21 (DE3) cells in log phase growth with the addition of 0.1 mM isopropyl β-D-1-thiogalactopyranoside (IPTG) and subsequently incubated with shaking at 20 °C for 20 h. Cells were harvested by centrifugation, resuspended in 20 mM Tris HCl, 150 mM NaCl, 20 mM imidazole (pH 7.5), and lysed by sonication. Soluble protein was purified by standard nickel affinity chromatography methods, 3C protease was added at a ratio of 1:100 (wt/wt) to remove the hexahistidine tag, and proteolysed material isolated by a second nickel affinity chromatography purification. Sgo_R10 and Sgo_R3-4 were further purified by preparative SEC using a Superdex 75 16/600 column (GE Healthcare) equilibrated in 20 mM Tris HCl, 150 mM NaCl (pH 7.5) (Sgo_R10) and (pH 8) (Sgo_R3-4). Sgo_R2-8 constructs were transformed into Rosetta-2 *E. coli* (DE3) cells and grown in LB-Miller media containing 100 μg/mL ampicillin at 37 °C to an optical density (OD)_600_ of 0.5 to 0.6. Gene expression was induced by addition of 0.1% (wt/vol) L-arabinose, followed by incubation at 20 °C. Soluble protein was purified as described for Sgo_R10. Protein-containing cysteine mutations were purified by affinity chromatography and SEC with the addition of 5 mM β-mercaptoethanol at all steps. Protein samples prepared for SEC-SAXS were dialyzed into 20 mM Tris HCl, 150 mM NaCl, 1 mM EDTA (pH 7.5).

### Protein Crystallization.

Proteins were concentrated (Δ*N*-Sgo_R2 to 48 mg/mL; Sgo_R10 to 30 mg/mL and Sgo_R3-4 to 35.2 mg/mL) by centrifugal filtration with 3 kDa molecular weight cutoff (MWCO) PES (Vivaspin). Δ*N*-Sgo_R2 crystallized by sitting drop vapor diffusion within 9 mo in conditions comprising 0.1 M Hepes (pH 7), 2.4 M ammonium sulfate at 291 K. This crystal was passed through 4 M sodium malonate prior to flash cooling. Sgo_R10 crystallized in 5 wk at 277 K in conditions comprising 2.2 M ammonium sulfate and 150 mM potassium thiocyanate. The crystal was harvested under mineral oil and flash cooled in liquid nitrogen prior to data collection. Sgo_R3-4 crystallized in 4 d in conditions comprising 65% (vol/vol) 2-methyl-2,4-pentanediol and 0.1 M Tris HCl (pH 8) at 277 K and flash cooled in liquid nitrogen prior to data collection.

### Structure Determination.

X-ray data were collected at 100 K on the I03 beamline at Diamond Light Source (Didcot, UK) using a Pilatus 3 6M detector. Data were indexed and integrated by XDS (38) and scaled and merged by Aimless (39). The structure of Δ*N*-Sgo_R2 was solved by MR with 2 antiparallel 5 residue ideal β-strands using Fragon (23), and the model was built automatically with ARP/wARP (40). Sgo_R3-4 and Sgo_R10 were phased by molecular replacement with Phaser (41) using search model Δ*N*-Sgo_R2. Models were manually built using Coot (42) and refined to completion with REFMAC5 (43) for Sgo_R3-4 and PHENIX (44) for Sgo_R10 (Table 1). The coordinates and structure factors have been deposited in the Protein Data Bank with accession codes (Δ*N*-Sgo_R2, 7AVJ; Sgo_R10, 7AVK; Sgo_R3-4, 7AVH). The structures were aligned by secondary structure matching with Superpose (45) and cartoons rendered with CCP4mg (46) with secondary structure defined by the database of protein secondary structure assignment (DSSP) (47).

### Determination of *T*_m_.

Differential scanning fluorimetry (DSF) was peformed using a Nanotemper Prometheus NT.48 instrument. Protein concentrations were 1 mM, and solution conditions were 20 mM Tris HCl (pH 7.5), 150 mM NaCl.

### SAXS.

SAXS experiments were performed at beamline B21, Diamond Light Source (Didcot, UK) over a momentum transfer range (*q*) of 0.01 Å^−1^ < *q* < 0.4 Å^−1^. Scattering intensity (*I* versus *q*, where *q* = 4πsinθ/λ and 2θ is the scattering angle) was collected using a Pilatus 2M detector, with a beam-to-detector distance of 4,014 mm and an incident beam energy of 12.4 keV. Sgo_R3-4 and Sgo_R2-8^S666C/S1086C^ were injected on an inline Shodex KW-203.5 column equilibrated in 20 mM Tris⋅HCl, 150 mM NaCl, 3 mM KNO_3_, 5mM β-mercaptoethanol (pH 7.5), each at 7.5 mg/mL, and data processing and reduction was performed with Chromixs (48). *R*_g_^c^ (Ln[*I*(*q*) versus *q*^2^] and Distance Distribution (*P*(*r*)) plots were calculated with Primus (49). The range of useful scattering angles was assessed using Shanum (50). SWISS-MODEL (51) was used for the generation of a structural model of Sgo_R2-8, using the Sgo_R3-4 crystal structure to generate iterative tandem domains for R3-5, R5-6, and R7-8. Sgo_R2 of the Sgo_R2-8 model was generated based on Sgo_R4 from the Sgo_R3-4 structure. For validation of the Sgo_R3-4 crystal structure, tag residues unresolved in the crystal structure were added using Modeler and all-atom ensembles generated using Allosmod (51). In each case, 50 independent pools of 100 models were created, and calculation and fitting of theoretical scattering curves to experimental data were performed using FoXS (52). This process was automated using Allosmod-FoXS (53). Plots were generated using OriginPro version 9.5.5.409 (OriginLab), as were the gradients of the linear regions of double logarithmic plots for the calculation of Porod exponents (54).

### Production of Fluorescently Labeled Protein.

As described above, cysteine residues were engineered into the third and eighth repeats of the Sgo_R2-8 construct at positions (S666, S1086), where fluorescence quenching by nearby amino acid residues is minimized. Sgo_R2-8^S666C/S1086C^ (27 μM) was dialyzed into 150 mM NaCl, 20 mM Tris HCl, 1 mM EDTA (pH 7.5), followed by dialysis into 150 mM NaCl, 20 mM Tris HCl, 1.35 mM Tris(2-carboxyethyl)phosphine (TCEP) (pH 7.5). The protein was then reacted at 20 °C in low-light conditions with a 20× molar excess of Alexa Fluor 488 C_5_ maleimide (ThermoFisher, 540 μM), which was added step-wise over a period of 1.5 h using a 10 mM stock in anhydrous DMSO. The labeling reaction was quenched by adding dithiothreitol (DTT) at 10× molar excess to the maleimide. The protein solution was dialyzed into 150 mM NaCl, 20 mM Tris HCl, 1 mM DTT (pH 7.5) prior to purification by SEC on a S200 30/10 column (Amersham) equilibrated in the same buffer to remove remaining free dye. Purified proteins were stored at −80 °C. The labeling efficiency (∼2 fluorophores/protein) was estimated from the spectrophotometrically determined concentrations of fluorophore (ε_495__nm_ = 72,000 M^−1^ ⋅ cm^−1^) and protein (ε_280__nm_ = 99,475 M^−1^ ⋅ cm^−1^) after correction for absorption at 280 nm by the fluorophore.

### Sample Preparation for SHRImP-TIRF Microscopy.

A 100-mM Trolox solution was freshly prepared by solubilizing 25 mg of Trolox powder (Fluka) in 50 μL methanol, followed by dilution with 850 μL of 0.31 M NaOH solution. All stock buffer solutions were passed through a 0.22-μm pore filter. Adsorption buffer contained 10 mM Hepes, 10 mM NaCl (pH 7.0), 1 mM Trolox, 0.02% (wt/vol) 5-μm diameter silica beads (Bangs Labs), 8 pM Alexa Fluor 488 (AF488)-labeled Sgo_R2-8^S666C/S1086C^ protein, and, in the “blocked” samples only, 800 pM unlabeled Sgo_R2-8 protein. Imaging buffer contained 10 mM Hepes, 10 mM NaCl (pH 7.0), and 1 mM Trolox. Poly-D-lysine-coated quartz slides were prepared as described previously (16). A total of 1 μM AF488-Sgo_R2-8^S666C/S1086C^ and 200 nM Sgo_R2-8 stock solutions were thawed from −80 °C storage and diluted with 20 mM Tris HCl, 150 mM NaCl (pH 7.5) buffer to the desired concentration before addition to the adsorption buffer at a 25× or 50× dilution. In “blocked” samples, the molar concentration of unlabeled Sgo_R2-8 in the adsorption buffer was maintained at 100× the molar concentration of AF488-Sgo_R2-8^S666C/S1086C^.

In low-light conditions, 50 μL of adsorption buffer was distributed along the center line of a 2 μg/mL or 20 μg/mL poly-D-lysine-coated quartz slide and then covered with a clean coverslip (No. 1, 22 mm × 64 mm, Menzel-Gläser). A flow chamber was created by sealing the two opposite, short sides of the coverslip with nail varnish. A small amount of imaging buffer was added along the unsealed sides of the flow chamber to prevent it from drying out. After 10 min of incubation at room temperature (20 to 22 °C), ∼500 μL of imaging buffer was flowed through the chamber created by the slide-silica bead-coverslip sandwich to wash away unbound protein, and the chamber was sealed with nail varnish.

### SHRImP-TIRF Microscopy.

Fluorescence excitation and detection of AF488 dye emission was achieved using a custom, prism-coupled TIRF microscope as described previously (16) with the following modification and increased optical magnification. Quantum dots were not routinely added to the adsorption buffer as an image-focusing aid. Video data (100 frames) were collected using an Evolve 512 (Photometrics) electron-multiplying CCD camera (500 ms exposure) and the pixel size was equivalent to 96 nm in the magnified image.

### Detection and Localization of Single Fluorophores.

Fluorescent spot detection and calculation of inter-AF488 dye distances for each spot that photobleached in two steps were performed as previously described (16). In addition, an eccentricity ratio was calculated for each spot (using the intensity profile in the *x* and *y* directions) to remove events that included weakly surface-adsorbed AF488-labeled protein or partially photobleached clusters of AF488-labeled protein. The intensity profile was calculated for a central region (2 × 10 pixels^2^) along the *x* and *y* axes of each spot image. This image was the sum of the first 5 video frames for each 10 × 10 pixel^2^ image stack (100 frames). The *x* and *y* intensity profiles were fit with a one-dimensional Gaussian function in MATLAB (MathWorks), and a ratio of the widths for the Gaussian fits (= σ _*y*-axis_/σ _*x*-axis_) was used to obtain the spot eccentricity. Fluorescent spots with an eccentricity ratio between 0.9 and 1.1 were retained (this filter removed 15 to 30% of the spots in an experiment). Bin size for the inter-AF488 dye distance histograms was calculated using the Freedman-Diaconis rule (55), and a single Gaussian distribution was fit to each histogram in KaleidaGraph (Synergy Software).

### Molecular Dynamics Simulations.

Molecular dynamics simulations were performed starting from the X-ray crystal structure model of Sgo_R3-4. Simulations have been performed using the CHARMM36m (56) force field and NAMD (57). The protein was energy minimized and solvated in a periodic rectangular box 123 Å × 46 Å × 40 Å needed to guarantee a layer of at least 12 Å solvent around the elongated protein. After a 1-ns equilibration, the systems were simulated at 303 K for 0.8 μs. Simulations were performed in the isothermal-isobaric ensemble, where the temperature was kept constant on average through a Langevin thermostat and the pressure was set to 1 atm through an isotropic Langevin piston manostat. For each saved frame of the simulation (one every 2 ns), the positions of the Ca atoms of Sgo_R4 were least square superposed to those of Sgo_R3, which implies a translation and a rotation around the each of the three principal axes of Sgo_R3 (hence applying the reverse transformation to the coordinates of Sgo_R4 the original conformation of that frame is recovered). Constructs with N domains were obtained by taking the original X-ray crystal structure, duplicating the coordinates of Sgo_R4 and applying to them the reverse transformation (using translation and rotations from random frames of the trajectory) N times.

### Periscope Protein Identification in NCTC3000 Collection.

A total of 2,579,577 proteins were extracted from 734 annotated bacterial genomes downloaded from the NCTC3000 project website (October 2019). Tandem repeats longer than 50 residues and with at least 80% sequence identity were detected with the T-REKS tool. Repeat sequences were clustered using BLASTp with a bit score threshold of 30 and extracting connected components of the resulting sequence similarity network (SSN). Similarly, proteins containing the long repeats were clustered using BLASTp with a bit score threshold of 100 and additional minimum sequence identity of 90% and coverage of 50% thresholds, by extracting the connected components of the SSN. Proteins were mapped to UniProt (58) identifiers (version 2020_04), with their associated Gene Ontology (GO) terms, and to Pfam (59) families (version 32.0) using PHMMER. Several new Pfam families were created from repeat sequences during the course of this study, including SHIRT (PF18655), YDG (PF18657), MBG_2 (PF18676), SSSPR-51 (PF18877), CshA_repeat (PF19076), and Big_13 (PF19077), among others. Phylogenetic trees of bacterial genomes were generated by first selecting all pairs of homologous genes for each genome pair using BLASTn, then computing a genomic sequence identity matrix, and finally creating a dendrogram of strains by hierarchical clustering.

### Analysis of Interdomain Linkers and Domain Termini Orientation.

A subset of 11 proteins with diverse domain repeats were selected from the Periscope Proteins table. Interdomain linkers were extracted from the protein sequences according to the Pfam domain boundaries for each family, with minor adjustments to cover the structural boundaries for incomplete Pfam models (missing terminal residues). Structures of the domain repeats, or close homologs from the same family or families within the same Pfam clan, were selected for each protein. The distance between the terminal residues and their relative orientation were calculated using the TADOSS software (60). The distance is measured as the Euclidean distance between the C-alpha atoms of the N-terminal and C-terminal residues, while the orientation is measured as the angle between the vectors formed by the four N- and four C-terminal residues (C-alpha atoms).

## Supplementary Material

Supplementary File

Supplementary File

## Data Availability

Atomic models of Δ*N*-Sgo_R2, Sgo_R10, and Sgo_R3-4 have been deposited in the PDB with IDs 7AVJ, 7AVK, and 7AVH, respectively. All other study data are included in the article text and supporting information.
