# Supplementary material for: Periscope Proteins are variable-length regulators of bacterial cell surface interactions
Source: Proc Natl Acad Sci U S A. 2021 May 31;118(23):e2101349118. doi: 10.1073/pnas.2101349118 (PMC8201768; doi:10.1073/pnas.2101349118)
Supplement: Supplementary File [file pnas.2101349118.sapp.pdf]

This PDF file contains:

Figures S1 to S6

Tables S1 to S3

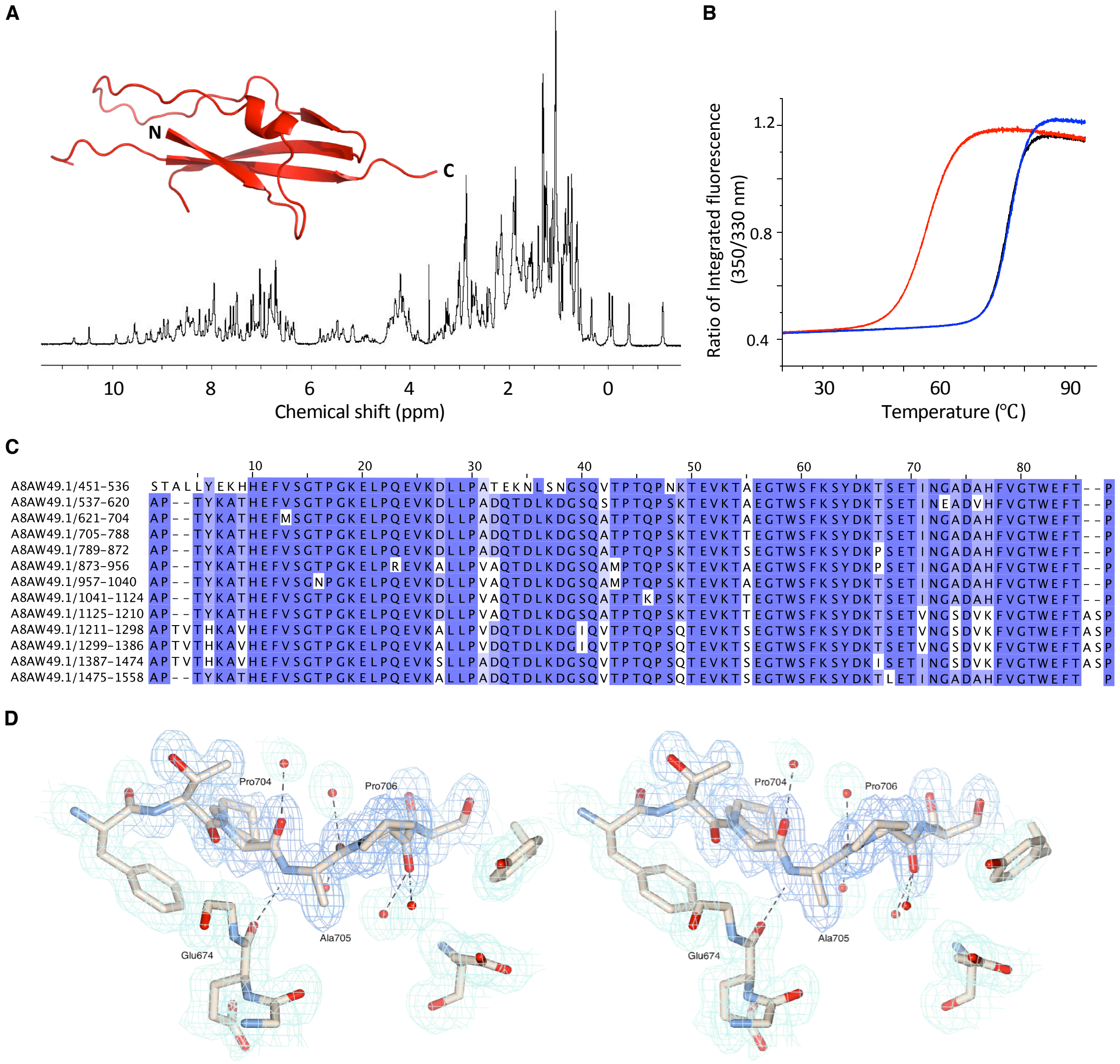

Fig. S1. Defining the structural repeats of Sgo_0707 from *Streptococcus gordonii.*

(*A*) 1D ^{1}H NMR spectrum of ΔN-Sgo_R2, structure of ΔN-Sgo_R2 inset. (*B*) T_m values for ΔN-Sgo_R2 (red), Sgo_R3 (black) and Sgo_R10 (blue) determined using nanoDSF. (*C*) sequence alignment of the 13 adjacent SHIRT domain repeats in Sgo_0707 protein. (*D*) Stereo image of

Sgo_R3-4 double repeat linker region Pro704-Ala705-Pro706 and flanking Thr703 and Thr707 (electron density $2mF_o$-DF_c map, blue chickenwire contoured at 0.1182 electrons/Å^3), illustrating that Ala705-Pro706 are solvent accessible with minimal contact with folded domains (hydrogen bonds, black dashed lines; water molecules, red spheres). Residues within 4 Å of the linker, and hydrogen bonded water molecules electron densities are rendered with cyan chickenwire contoured at 0.1182 electrons/Å^3. Consistent with the inter-domain linker having similar rigidity to the flanking SHIRT domains, temperature (B-factors) do not vary significantly across this region of the structure (average protein B-factor 18 Å^2; average B-factor linker residues 704—706 18.9 Å^2)

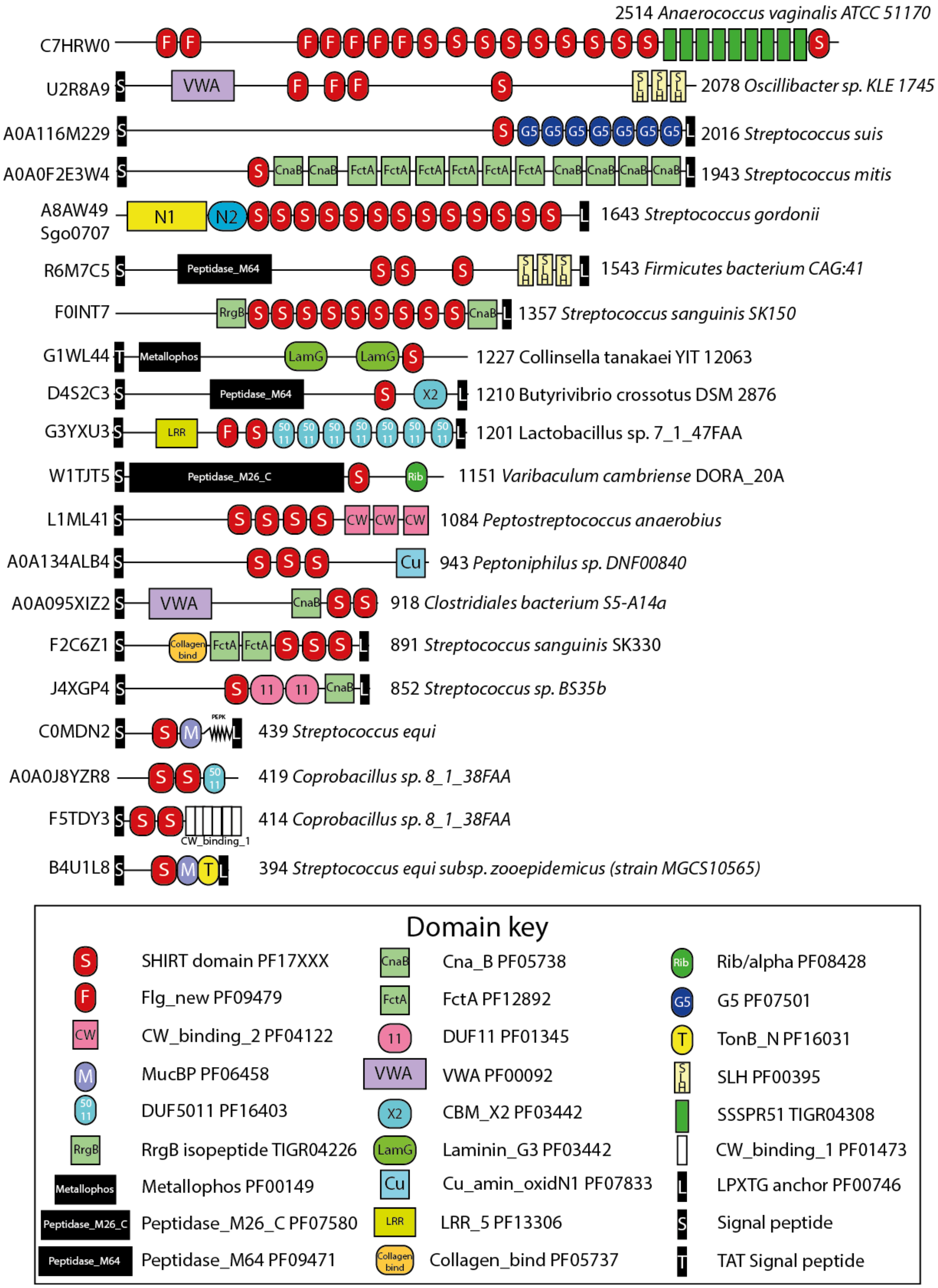

Fig. S2. SHIRT domains are found in many other proteins, often in tandem array.

Each line in the figure shows a representative protein domain architecture containing SHIRT/S domains with UniProt accession number shown at the left. The domain architecture shows boxes and shapes (Domain key) representing various domains, repeats and motifs identified in the proteins using Pfam and TIGRfams. The N1 and N2 domains from Sgo_0707 are included based on the known structure but are not represented within any domain database. Potential enzymatic domains are shown as the larger black boxes with white text, while the localization motifs are shown as small block boxes with a white letter to represent their type. At the end of each line the length of the protein is shown followed by the species and strain if known.

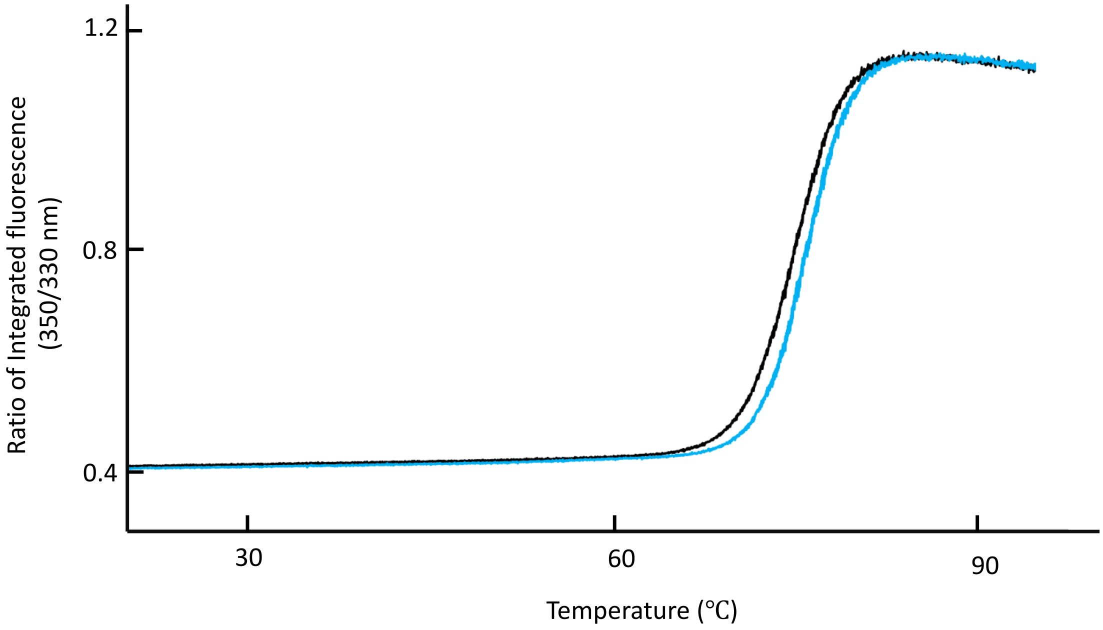

Fig. S3. The interdomain interface in Sgo_R3-4 is very limited.

T_m analysis using nanoDSF of Sgo_R3 (black) compared with Sgo_R3-4 (cyan).

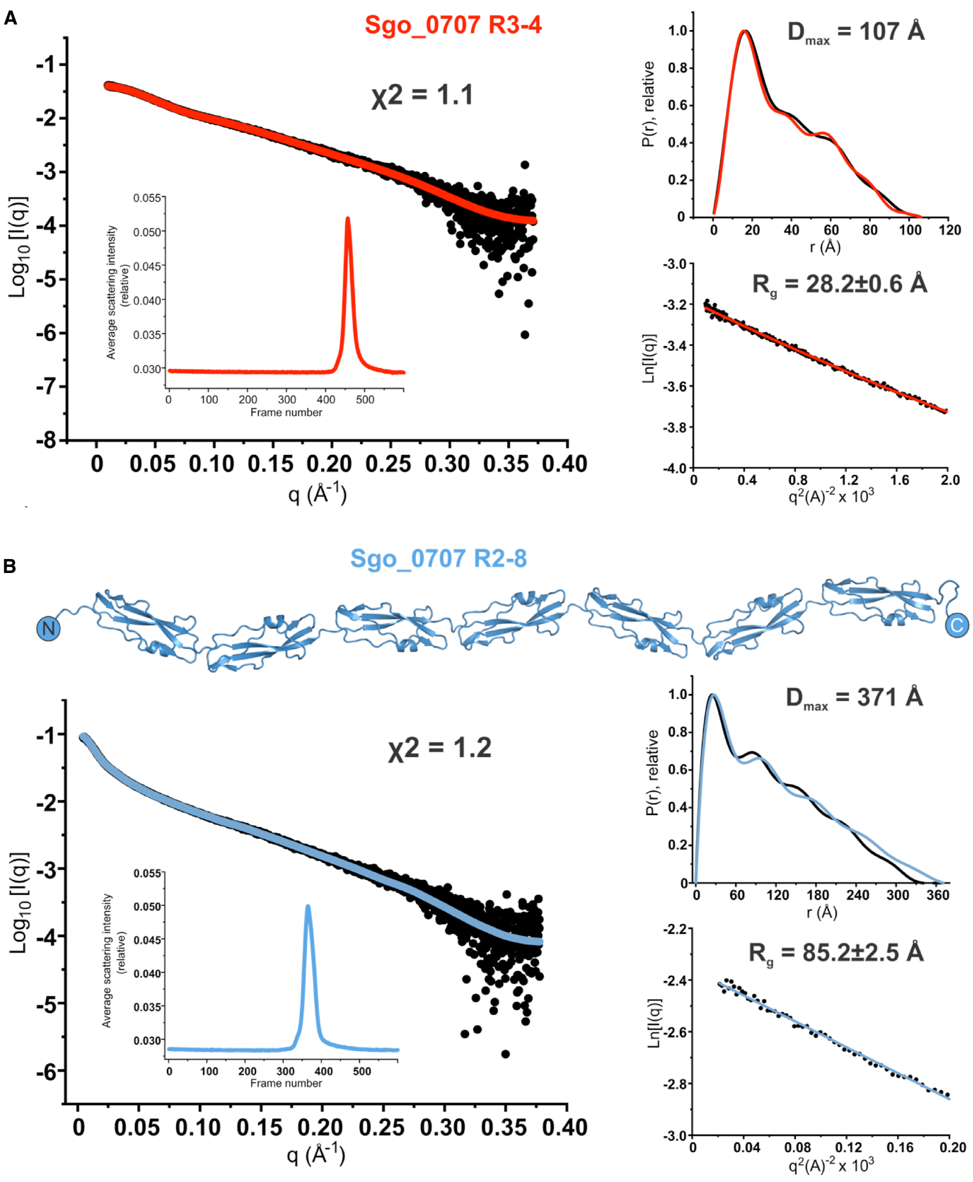

Fig. S4. Validation of Sgo_0707 R3-4 and Sgo_0707 R2-8 models in solution via SAXS.

(*A*) (Left) Fit of Sgo_0707 R3-4 crystal structure (see Fig. 2*A*) to experimental scattering data. Experimental (black) and model (red) scattering curves are displayed to a maximum scattering angle of q = 0.378 $Å^{-1}$. Scattering data are accounted for by a single Sgo_0707 R3-4 model, with fit value ($\chi 2$) displayed. A trace of the in-line SEC run is displayed (inset). Normalised pair-distance distribution function (experimental – black, model – red) is displayed, with the derived maximum intra-particle distance (D_{max}) displayed (real space R_g = 30.1±0.1 Å, *I*(0) = 0.04) (top right). The

linear portion of the Guinier region confirms monodispersity (q^*R_g range = 0.4–1.2; $I(0)$ = 0.04) (bottom right). (*B*) Fit of Sgo_0707 R2-8 model to experimental scattering data. A model for Sgo_R2-8 was generated using iterative placement of the Sgo_R3-4 crystal structure (top; see methods). Experimental (black) and model (blue) scattering curves are displayed to a maximum scattering angle of q = 0.368 Å^{-1} (left). Scattering data are accounted for by a single Sgo_R2-8 model, with fit value displayed. A trace of the in-line SEC run is displayed (inset). Normalised pair-distance distribution function (experimental – black, model – blue) is displayed, with the calculated D_{max} displayed (real space R_g = 96.2±0.5 Å, $I(0)$ = 0.10) (middle right). The Guinier region confirms monodispersity (q^*R_g range = 0.5–1.3; $I(0)$ = 0.093) (bottom right).

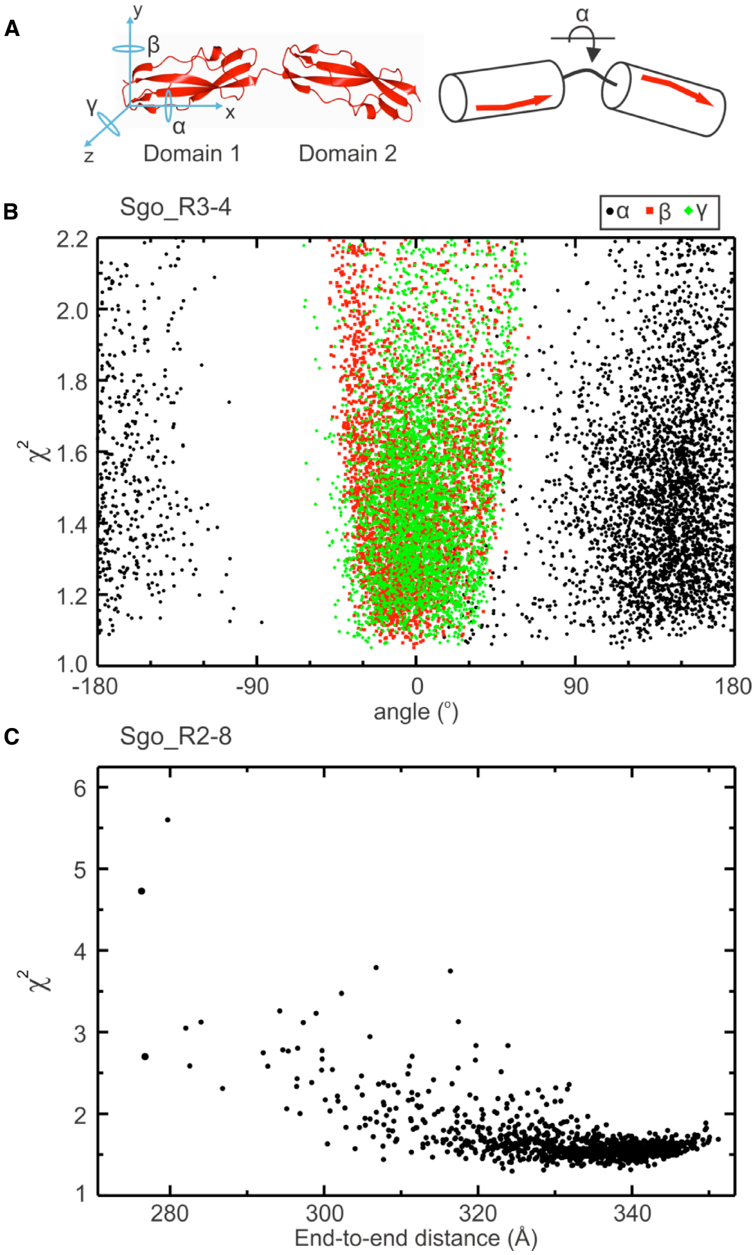

Fig. S5. Elongation of Sgo_0707 is dependent on rotation angle α.

(*A*) Definition of the three rotation angles (α, β, γ) between consecutive Sgo_0707 domains, along the axes x-, y- and z- (left). Domains can rotate around angle α to maintain elongation (right). (*B*) Fit of simulated Sgo_R3-4 models with variation of all three angles α, β, γ to experimental SAXS data. (*C*) Fit of simulated Sgo_R2-8 models to experimental SAXS data. End-to-end distance is defined as in Fig. 3.

A

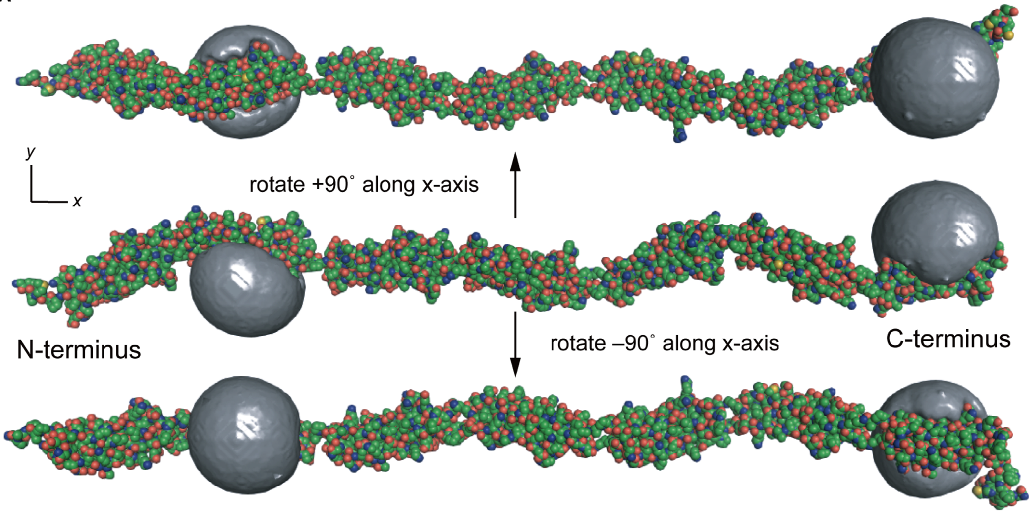

B

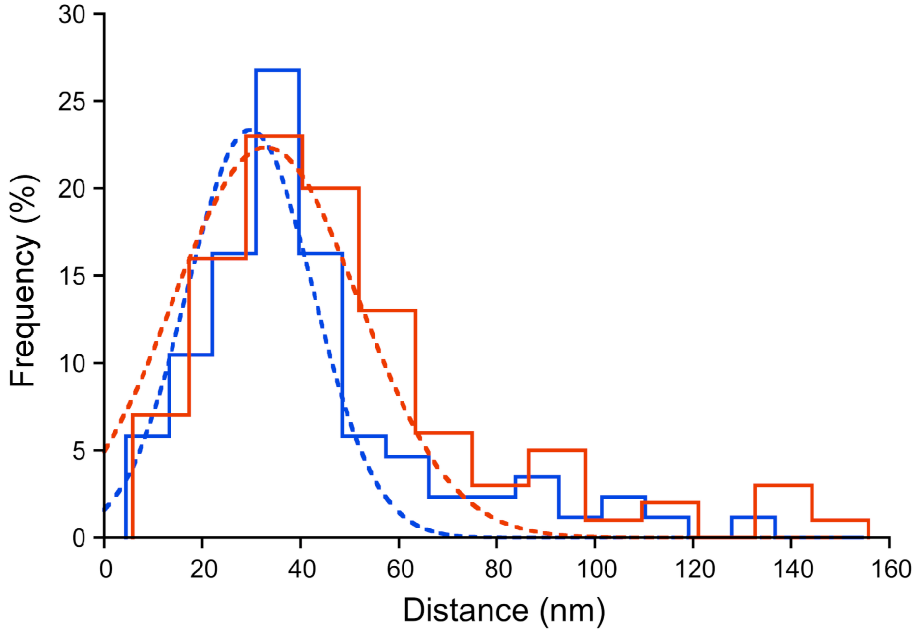

Fig. S6. Determination of inter-dye distances using SHRImP-TIRFM.

(*A*) The structural schematics show the accessible volume (AV) for both Alexa Fluor 488 (AF488) dyes attached to Sgo_R2-8$^{S666C/S1086C}$ via a C_5 linker at positions S666 and S1086. The N- and C-terminal ends of the Sgo_R2-8$^{S666C/S1086C}$ construct are labelled. The SAXS-derived structural model (*SI Appendix*, Fig. S4*B*) was utilised to simulate the AVs (grey envelopes) as described in the Methods section using three radii to estimate the volume of the AF488 dye. In this structural

model, the distance between the dye attachment points ($C_ß$ atoms of S666 and S1086 residues) is 24.0 nm, and the predicted distance between the mean dye positions is 24.1 nm. Images were prepared using the PyMOL Molecular Graphics System (version 2.4.0, Schrödinger LLC). (*B*) SHRImP-TIRFM determination of the inter-dye distances for AF488-labelled Sgo_R2-8$^{S666C/S1086C}$ immobilised on 2 µg/mL (blue) or 20 µg/mL (red) poly-D-lysine treated quartz surfaces in the presence of 100-fold molar excess of unlabelled blocking Sgo_R2-8 protein. Colour-coded, dashed lines indicate Gaussian fits to the histograms for the 2 µg/mL (n = 86, R^2 = 0.92) and 20 µg/mL (n = 100, R^2 = 0.94) poly-D-lysine-treated quartz surfaces (mean ± standard error = 29.8 ± 1.38 nm and 33.0 ± 1.90 nm, respectively).

Table S1: Primer sequences for SHIRT domain construct cloning and mutagenesis

**Construct**	**Primers**
ΔN-Sgo_R2 (aa 544–627)	5'-TCCAGGGACCAGCA**ATG**CATGAATTTGTGAGCGGCAC-3' and 5'-TGAGGAGAAGGCGCGTTAGGTGGCTTTGTAGGTCGG-3'
Sgo_R3 (aa 621–705)	5'-TCCAGGGACCAGCA**ATG**GCCCCGACCTACAAAGCCACC-3' and 5'-TGAGGAGAAGGCGCGTTAGGCCGGGGTAAATTCCCAGGTGC-3'
Sgo_R10 (aa 1211–1299)	5'-TCCAGGGACCAGCA**ATG**GCTCCAACAGTGACTC-3' and 5'-TGAGGAGAAGGCGCGTTATTAAGCTGGGCTCGCTG-3'
Sgo_R3-4 (aa 621–789)	TCCAGGGACCAGCA**ATG**GCCCCGACCTACAAAGCCACC-3' and 5'-TGAGGAGAAGGCGCGTTATGCCGGGGTGAATTCCCAGGTAC-3'
Sgo_R2-8 S666C	5'-GACTCAACCTTGTAAAACAG-3' and 5'-CTGTTTTACAAGGTTGAGTC-3'
Sgo_R2-8 S1086C	5'-GACAAAACCCTGTAAGACCG-3' and 5'-CGGTCTTACAGGGTTTTGTC-3'

Table S2: Synthetic DNA sequences used in this study

Sgo_0707 residues 544–795	catgaatttgtgagcggcaccccgggcaaggaactgcctcaggaagtgaaggacctgctgccg<br>gccgatcaaaccgatctgaaagacggcagtcagagcaccccgacccagccgagtaaaaccgag<br>gtgaaaaccgccgaaggcacctggagctttaagagctacgacaagaccagcgaaaccatcaac<br>gaagccgatgtgcactttgtgggtacctgggagttcaccccggccccgacctacaaagccacc<br>catgagttcatgagcggcacaccgggtaaagaactgcctcaagaggtgaaggatctgctgcct<br>gcagatcaaaccgacctgaaggatggcagtcaagccaccccgacacagccgagcaaaacagaa<br>gtgaagacagccgagggcacctggagcttcaaaagctatgacaaaaccagcgagaccattaac<br>ggcgcagatgcccactttgtgggcacctgggaatttaccccggccccgacatacaaggccacc<br>cacgagtttgtgagcggtacaccgggcaaagaactgccgcaggaagttaaagatctgctgccg<br>gccgaccagaccgacctgaaagatggtagccaggcaaccccgacccaaccgagcaagacagaa<br>gtgaaaaccaccgaaggcacctggagcttcaagagttatgataagaccagcgaaaccattaat<br>ggcgccgatgcccattttgttggtacctgggaattcaccccggcaccgacctataaggccacc
Sgo_R2-8	gcgccaacctacaaggcaactcacgagtttgtcagcggaactccaggaaaagaacttcca<br>caagaagtgaaggacctgcttccagcagaccaaacagacttgaaagatggtagtcaatcg<br>actccaacgcaaccaagtaaaaccgaggttaagacagcagaaggcacatggagcttcaag<br>tcctatgacaagacttccgaaaccatcaatgaagcagacgtacacttcgtaggaacatgg<br>gaattcaccccagcgccaacctacaaggcgactcatgagtttatgagtggaaccccaggt<br>aaagagcttccacaagaagtgaaagacctgcttccagcagaccaaacagacttgaaagat<br>ggaagccaagcgactccaactcaaccaagtaaaacggaagttaagacagcagaaggcact<br>tggagtttcaagtcatacgacaagacttctgaaaccattaatggcgcggacgcacacttc<br>gtaggcacatgggaattcaccccagcgccaacctacaaggcgactcatgagtttgtgagt<br>ggaaccccaggtaaagagcttccacaagaagtgaaagacctgcttccagcagaccaaaca<br>gacttgaaagatggaagtcaagccactccaacgcaaccaagtaaaacggaagtgaagacg<br>acagaaggtacttggagtttcaagtcatacgacaagacttctgaaaccattaatggcgcg<br>gacgcacacttcgtaggcacatgggaattcaccccagcgccaacctacaaggcgactcat<br>gagtttgtgagtggaaccccaggtaaagagcttccacaagaagtgaaagacctgcttcca<br>gcagaccaaacagacttgaaagatggaagccaagcgactccaacacaaccaagtaaaacg<br>gaagttaagacgtcagaaggcacttggagcttcaagtcctatgacaagccgtctgaaacc<br>atcaatggagcagacgcacacttcgtaggcacctgggaattcaccccagcgccaacctac<br>aaggcgacacacgagtttgtcagcggaactccaggcaaagagcttccacgagaagtaaaa<br>gcactgcttccagtagctcaaacagacttgaaagatggtagccaagcgatgccaacgcaa<br>ccaagtaaaacagaggttaagacagcagaaggcacttggagcttcaagtcctatgacaag<br>ccgtctgaaaccatcaatggagcagacgcacactttgtcggtacttgggaatttacccca<br>gcaccaacctacaaggcaacacacgagtttgtgagtggaaatccaggtaaagagcttcca<br>caagaagtaaaagatctgcttccagtagctcaaacagacttgaaagatggtagtcaagcg<br>atgccaacgcaaccaagtaaaacggaagttaagacagcagaaggtacttggagtttcaaa<br>tcatacgataagacttccgaaaccatcaatggagcagacgcacactttgtaggaacatgg<br>gaattcaccccagcgccaacctacaaggcgacacacgagtttgtcagcggaactccaggc<br>aaagagcttccacaagaagtaaaagatctgcttccagtagctcaaacagacttgaaagat<br>ggtagccaagcaacaccaacaaaaccaagtaaaacggaagtgaagacaactgaaggcact<br>tggagtttcaaatcatacgataagacttccgaaaccatcaatggagcagacgcacacttt<br>gtaggaacatgggaattcaccccagcg
Sgo_R10	gctccaacagtgactcataaagcagttcacgaatttgtgagtggaactccaggcaaagag<br>cttccacaagaagtgaaagccctgcttccagtagatcaaacagatctgaaagatggcatt<br>caagtgactccaacacaaccaagtcaaacagaggttaagacatcagaaggcacatggagc<br>ttcaagtcatacgataagacttcagagactgtcaacggttcagatgttaagtttgtagga<br>acatgggaatttacagcgagcccagcttaa

Table S3: Inter-domain linker and domain repeat termini orientation for a subset of 11 Periscope proteins. Columns indicate, from left to right: protein name, UniProt identifier, Pfam identifier of the domain repeat, inter-domain linker sequence or sequences if multiple linkers are observed (comma separated), PDB identifier of the domain repeat structure, Pfam identifier of the domain structure (asterisk indicates the use of a similar domain from the same Pfam clan), distance between the N- and C-termini of the domain structure, and angle between the N- and C-termini of the domain structure. The structure of the CshA_repeat (PDB: 6SZC) has not been used due to its incomplete N-terminus.

Protein	UniProt	Domain repeat	Inter-domain linker	PDB	Domain	NC-distance (Å)	NC-angle (rad)
SasG	Q2G2B2	G5-E	GP, GG	3TIQ	G5	70.54	2.24
				3TIQ	SasG_E	40.63	2.59
SAP077A_019	D2JAN8	Big_6	TTA	2YN5	Big_13*	36.93	2.57
Rib R4	P72362	Rib	DPR	6S5X	Rib	39.27	2.10
Sgo_0707	A8AW49	SHIRT	TPAPT, TASPAPTVT	7AVK	SHIRT	42.86	3.01
CshA	A0A0F2D244	CshA_ repeat	STE, RPE, TPT				
CdrA	A0A485CMM4	MBG_2	PAQ, KAL	4NG0	MBG*	39.06	3.09
E0F69_00680	A0A5S4TIX9	MucBP	ARQPLG, KQAQPLG	2KT7	MucBP	29.03	2.98
Spa	M4MB15	B	QAPKA	4NPF	B	31.70	1.70
Spg	A0A4V0F6C4	GA-like	ESAKKARISEATDGLSDF LKSQTPAE	2J5Y	GA-like	35.27	2.86
LytD	A0A2X2YDI6	SH3_3	TQTPEKPSTPESTEKTG	2KT8	SH3_3	7.39	0.94
Autolysin	P37710	LysM	GNTGGSGSGGSNNN	2MKX	LysM	5.01	0.60

Other supplementary materials for this manuscript include the following:

Dataset S1. Table of Periscope proteins identified from the NCTC3000 dataset. Columns names stand for: cluster identifiers (clustid), recognisable name from gene names and other literature references or UNK for unknown name (name), number of proteins in the cluster (size), minimum number of repeats (minrep), maximum number of repeats (maxrep), length of the repeat (replen), average percentage of protein sequence identity of repeats (psim), average percentage of DNA sequence identity of repeats (psim.dna), closest UniProt protein (uniprotid), Pfam families in the protein (pfam), name of Pfam families of the repeating sequence (pfam.rep.name), Pfam accession numbers in the repeating sequence (pfam.rep.acc), NCTC3000 species where the protein was found (species), and GO terms for location (GO_ location), function (GO_function) and process (GO_process).
